# Error in Commentary

**DOI:** 10.1001/jamanetworkopen.2026.1505

**Published:** 2026-02-16

In the Invited Commentary titled “Structural Approaches to Expand Access to Medication Treatment for Alcohol Use Disorder,”^1^ published on January 12, 2026, there was an error. In the fifth paragraph, rural counties should have been characterized as having a higher proportion of non-Hispanic White residents. This article has been corrected.^1^
